# Sliding of Proteins Non-specifically Bound to DNA: Brownian Dynamics Studies with Coarse-Grained Protein and DNA Models

**DOI:** 10.1371/journal.pcbi.1003990

**Published:** 2014-12-11

**Authors:** Tadashi Ando, Jeffrey Skolnick

**Affiliations:** Center for the Study of Systems Biology, School of Biology, Georgia Institute of Technology, Atlanta, Georgia, United States of America; NIDDK, National Institutes of Health, United States of America

## Abstract

DNA binding proteins efficiently search for their cognitive sites on long genomic DNA by combining 3D diffusion and 1D diffusion (sliding) along the DNA. Recent experimental results and theoretical analyses revealed that the proteins show a rotation-coupled sliding along DNA helical pitch. Here, we performed Brownian dynamics simulations using newly developed coarse-grained protein and DNA models for evaluating how hydrodynamic interactions between the protein and DNA molecules, binding affinity of the protein to DNA, and DNA fluctuations affect the one dimensional diffusion of the protein on the DNA. Our results indicate that intermolecular hydrodynamic interactions reduce 1D diffusivity by 30%. On the other hand, structural fluctuations of DNA give rise to steric collisions between the CG-proteins and DNA, resulting in faster 1D sliding of the protein. Proteins with low binding affinities consistent with experimental estimates of non-specific DNA binding show hopping along the CG-DNA. This hopping significantly increases sliding speed. These simulation studies provide additional insights into the mechanism of how DNA binding proteins find their target sites on the genome.

## Author Summary

DNA binding proteins efficiently search for their cognitive sites on long genomic DNA in cells to control biological activities. Recent experimental studies have revealed that the proteins use not only three-dimensional diffusion, but also one-dimensional diffusion (sliding) on DNA for this search process. For a better understanding of this biological process, we need to elucidate the mechanism of sliding. We report here molecular simulations using newly developed coarse-grained protein and DNA models for elucidating the nature of the sliding motions. Our simulation results show that: 1) hydrodynamic interactions between protein and DNA reduce sliding rate by 30%, 2) structural fluctuations of DNA give rise to steric collisions between proteins and DNA, which facilitate sliding motions, and 3) proteins with low binding affinities to DNA can hop along the DNA, resulting in a significant increase in sliding speed. These simulation studies provide additional insights into the mechanism of how DNA binding proteins find their target sites on the genome.

## Introduction

In living cells, DNA-binding proteins search for their specific target sites on DNA to initiate many biological processes, such as transcription, repression, activation, etc. How can proteins find their target sites on a long genome DNA? Many experimental and theoretical studies have been done over the past decade to address this issue (see review articles [1], [2] and references therein). The question as to how proteins find their DNA binding sites arose from the experimental observation that the association rate of the lactose repressor and its target site on DNA was significantly (about 100 times) higher than the expected Smoluchowski reaction rate in three-dimensional (3D) space [3]. To explain this discrepancy, Riggs proposed a mechanism that the protein does not rely on 3D diffusion alone for target search, but also non-specifically binds to the DNA at a random location, then undergoes one-dimensional (1D) Brownian diffusion (or sliding) along DNA to their target sites [3]. This is based on the idea of reducing the dimensionality of diffusion based reactions in biological systems as originally suggested by Adam and Delbeück in 1968 [4]. This mechanism, so-called “facilitated diffusion” or a “1D/3D mechanism”, was later expanded on by Berg, Winter, and von Hippel [5]. In their model, DNA binding proteins have three modes for target search: 1) 1D sliding on DNA without dissociation, 2) 1D hopping along the DNA via a series of microscopic dissociation and association events to a nearby location, and 3) jumping or diffusion in 3D for inter-segmental transfer [1], [2]. The significant facilitation of the rate that lactose repressor finds its DNA target site can be explained by an acceleration due to the electrostatic interactions between a positively charged site on the protein and the negatively charged phosphate groups in DNA at the low salt concentrations used in the experiment [6]. This classical experiment and the idea of facilitated diffusion have driven many experimental and theoretical studies for over a decade. Indeed, single molecule experiments have confirmed 1D sliding motions of several DNA binding proteins along DNA *in vitro* [7]–[12] as well as *in vivo* [13], [14].

Assuming that only 1D sliding and 3D diffusion are at play, a simple analytical analysis gives the association rate of a DNA-binding protein to its target site [1], [2]:
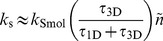
(1)where *k*_Smol_ is the Smoluchowski rate for random association to target site with 3D diffusion, 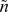 is the average distance in units of base-pairs (bp) moved during a single sliding step. *τ*_1D_ and *τ*_3D_ are the average durations of 1D sliding and 3D diffusion in one round of a 1D and 3D search, respectively. The Smoluchowski rate has the form *k*_Smol_ = 4π*D*_3D_ *bfN*_A_ with the diffusion coefficient of the protein in 3D, *D*_3D_, the spacing between the base-pairs of DNA is *b* ( = 3.4 Å), and the fraction of the protein's surface that contains the reactive binding interface, *f*, and *N*_A_ is Avogadro number. With the measured diffusion coefficients of proteins ranging from *D*_3D_≈(1–5)×10^−6^ cm^2^ s^−1^ and assuming *f*≈0.2–0.5, we obtain *k*_Smol_≈10^8^ M^−1^ s^−1^ [1], [2]. The sliding length can be written by 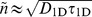 with a 1D diffusion coefficient *D*_1D_. This equation demonstrates that binding to a non-specific site on DNA slows down the search process by a factor of *τ*_3D_/(*τ*_1D_+*τ*_3D_) and this slow-down is compensated for by the sliding term 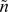. By setting *dk*_s_/*dτ*_1D_ = 0 and assuming *D*_1D_ is independent of *τ*_1D_, we found that the maximum rate constant is achieved if *τ*_1D_ = *τ*_3D_ [1], [2]. However, in a bacterial cell, the theoretical estimate for *τ*_3D_/(*τ*_1D_+*τ*_3D_) has range of 10^−1^–10^−4^ [1], which is verified by *in vivo* measurements [13]. This ratio is far from the optimal ratio in the theory. Additionally, 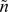 is estimated to be in the range of 10^2^–10^3^ bps *in vitro* [13] and is likely less *in vivo* [14]. Therefore, the 1D/3D mechanism does not allow for significant facilitation and may reduce the efficiency of the protein–DNA search in bacterial cells [1], [2], [15]. Possible reasons why 1D sliding does not increase the rate of diffusive motion may be related to spatial effects of the genome [1]. Thus, despite significant progress in theoretical and experimental approaches, the detailed mechanisms of protein motion along DNA and its biological role are still not well understood. What are the protein and DNA conformations, energetics, and search dynamics that enable efficient target search along genomic DNA in crowded intracellular environments? For better understanding of the search processes at molecular to cellular levels, simulation studies can play an important role for connecting theory and experiment.

Since DNA has a helical structure, one might expect that proteins rotate along the helical groves of the DNA during 1D sliding. Schurr first derived a theoretical expression for an apparent 1D diffusion coefficient of this rotation-coupled sliding by a non-specifically DNA bound protein along DNA based on hydrodynamic theory [16]. In his model, if the protein is approximated as sphere, the total friction is the sum of translational and rotational friction, in which it is assumed that the center of mass of the protein always remains on the DNA axis. Recently, Bagchi, Blainey, and Xie extended his model to take into account off-axis rotational diffusion of the proteins [17]. Their model, called the “BBX model”, is expressed by
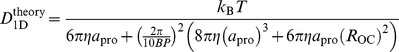
(2)where *k*_B_ is Boltzmann's constant, *T* is the temperature, *η* is the viscosity of water, *a*_pro_ is the radius of the protein, *BP* is the distance between two base pairs of DNA, equal to 3.4 Å, and *R*_OC_ is the separation between the protein center of mass and the longest axis of DNA. The first and second terms in the denominator describe translational friction along the longest axis and rotational friction on the axis, and are the same as in the Schurr's model. The third term in the denominator, an additional term in the BBX model, accounts for the friction associated with off-axis circular translational motion around the axis. This third term is essentially translational friction. The most important consequence of the BBX model is that a 1/(*a*_pro_)^3^ size dependence of diffusion is expected, in contrast to the usual Stokes-Einstein 1/*a*_pro_ dependence for pure translational motion. The same group experimentally measured the apparent 1D diffusion coefficients of various size transcription factors by a single molecule analysis *in vitro* and showed the 1/(*a*_pro_)^3^ dependence of the diffusion coefficients. This strongly suggests that proteins non-specifically bound to DNA undergo rotation-coupled sliding [7]. BBX theory predicts a 100–200-fold reduction in a protein's 1D diffusivities relative to their diffusivities in 3D for typical transcription factors modeled as spherical objects [17]. Interestingly, the diffusion coefficients of most transcription factors predicted by the BBX model are still 2–5 times larger than experimental values, which may be attributed to free energy roughness to sliding that arises from details of the protein-DNA interactions [7], [17]. Thus, on average diffusion constants on DNA for most transcription factors seems to be less than that expected for 3D diffusion in the absence of the DNA.

Apart from the theoretical analyses, molecular simulation studies also observe rotation-coupled sliding of DNA binding proteins [18]–[20]. In those studies, the dynamics of coarse-grained (CG) DNA and protein models were analyzed by molecular dynamics simulations in a simplified implicit solvent model. Proteins are modeled by strings of beads representing α carbon atoms of amino acids, with each nucleotide represented by a few beads. These studies provide molecular and atomistic views of the sliding process.

Here, we have developed a CG model for protein sliding along DNA. In our simulations, hydrodynamics interactions (HI) are considered. As is well known from polymer dynamics [21], HI can significantly alter the dynamics of macromolecules. Including HI in the simulations makes it possible to compare to the BBX theory of rotation-coupled sliding. We remind the reader that the BBX model assumes that 1) the protein is a sphere, 2) the protein follows a helical track along, and never detaches from, the DNA, 3) HI between the protein and DNA are ignored, and 4) there is no energy roughness along with sliding. Here, we would like to address the following questions: What are the effects of HI between the protein and DNA on 1D diffusion of proteins along DNA? How does DNA flexibility affect 1D sliding? These effects are difficult to handle in the theoretical analysis. We first build CG models of DNA binding proteins and DNA, and then estimate protein-DNA binding affinities in the CG model by an umbrella sampling method. Using the model, we then perform BD simulations under various conditions to answer these questions.

## Methods

### Brownian dynamics algorithm with hydrodynamic interactions

The BD simulations were performed using a second-order Runge-Kutta algorithm [22]. For constructing the 3*N*×3*N* diffusion matrix **D** of a given simulation system with *N* particles, we employ the Rotne-Prager-Yamakawa (RPY) tensor [23], [24], described by
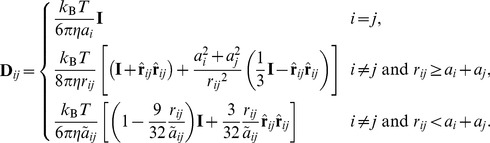
(3)Here, **D** *_ij_* is the 3×3 diffusion tensor for particles *i* and *j*, **r** *_ij_* is **r** *_i_*−**r** *_j_*, with the particle position vector **r**, *r_ij_* is the length of **r** *_ij_*, and 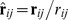, **I** is the 3×3 unit tensor, *k*_B_ is Boltzmann's constant, *T* is the temperature, *η* is the viscosity of water, and *a_i_* is the Stokes radius of particle *i*. 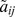 is an effective Stokes radius of the *i* and *j* particle pair. In this study, 
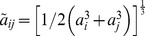
 was used [25].

### CG-DNA and protein models

**Figure 1:**
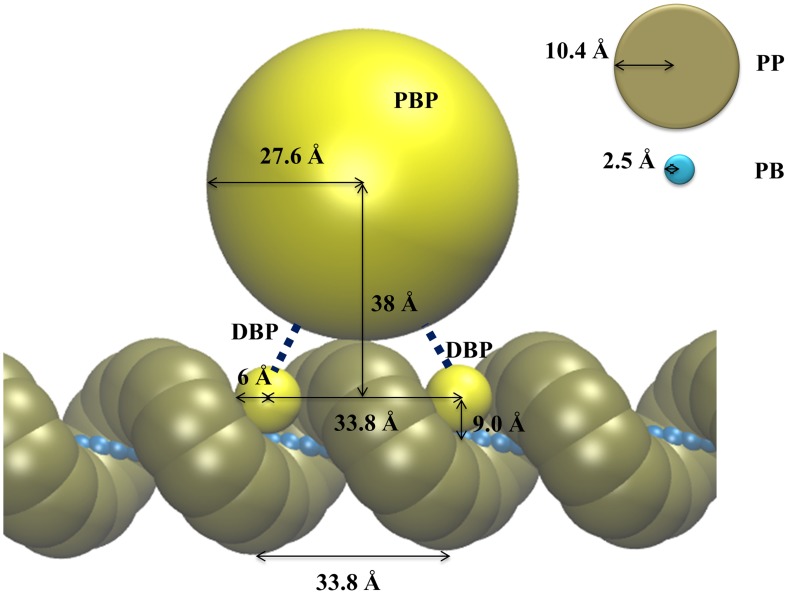
Schematic view of the CG-protein and CG-DNA models. PBP and DBP represent a *P*rotein *B*ody *P*ortion and a *D*NA *B*inding *P*ortion of the CG-protein model, respectively. PP and PB represent a *P*seudo *P*hosphate of two adjacent nucleotides and a *P*seudo *B*ackbone of double strand DNA, respectively. PBP and DBP beads are connected to each other by a harmonic potential, represented as a black line. The excluded volume radii for each bead used in the simulations are shown.

A schematic view of our CG protein and DNA model is shown in Fig. 1. Many DNA binding proteins form homo dimers, where the dimeric proteins have two DNA binding domains, e.g. lactose repressor, tryptophan repressor, λ repressor, etc. [26]. In this work, a protein molecule is represented by three beads: one representing a *p*rotein *b*ody *p*ortion, named the “PBP” bead, and the rest of the beads represent the *D*NA *b*inding *p*ortion, named “DBP”, which have positive charges to bind to DNA. For the DNA molecule, the two adjacent nucleotides in the double strand are represented by a *p*seudo *p*hosphate “PP” bead at the position of the phosphate atom in one strand of the canonical B-form of DNA. Since the bead represents two adjacent nucleotides, we set the effective charge of the PP beads to be −2. PP beads are connected to *p*seudo *b*ackbone “PB” beads, located on the long axis of the DNA. Radii, *σ*, for excluded volume effects explained below, Stokes radii, *a*, and effective charges, *q*, of the beads are listed in Table 1. *σ* values were determined to represent geometrical features of DNA. The distance between adjacent phosphate atoms is about 12.6 Å in B-DNA, with two adjacent nucleotides represented by one PP bead. To reproduce the excluded volume of the two adjacent nucleotides, the radii of the PP beads were set to 10.4 Å. The center of PBP was placed at 38 Å which gives an off-axis distance of 47 Å ( = 9+38) between *R*oc values of LacI (55 Å) and hOgg1 (25 Å) as reported in Ref. [17]. The radii of the PBP were set to 27.6 Å ( = 38–10.4 Å). DBP's radii of 6 Å were used to geometrically fit between PP beads. This is slightly smaller than the surface distance between PP beads, 7 Å. The assigned *a* for beads of CG-DNA give translational diffusion coefficients of small DNA fragments (8 bp to 24 bp) close to the experimental values [27].

**Table 1:** Names, radii, Stokes radii, and charges of particles in the CG model.

Bead name	Radius, *σ* (Å)	Stokes radius, *a* (Å)	Effective charge, *q*
PP (in DNA)	10.4	7	−2
PB (in DNA)	2.5	7	0
PBP (in protein)	27.6	30, 40, and 50	0
DBP (in protein)	6	8	Various

The pitch in B-form DNA is 33.8 Å, i.e. that of 10 base pairs, so that distance between adjacent PB beads is 3.38 Å and the torsion angle defined by PP(*α*) – PB(*α*) – PB(*α*+1) – PP(*α*+1) for the *α*-th pseudo residue is 36 degrees. The distance between PP(*α*) – PB(*α*) is 8.973 Å, which is the position of the phosphate atom from the longest axis of B-DNA. Adjacent beads, PP(*α*) – PB(*α*) and PB(*α*) – PB(*α*+1), are connected by a harmonic potential,
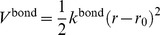
(4)where *k* ^bond^ is the force constant, and *r*_0_ is the equilibrium distance between beads making the bond. Beads in CG-protein model are also connected by Eq. (4). All bond distances and force constants are listed in Table 2.

**Table 2:** Parameters used in the bond energy of the CG model.

Bond type	*k* ^bond^ (kcal/mol/Å^2^)	*r*_0_ (Å)
PP(*α*) – PB(*α*)	100 *k*_B_ *T*/*r*_0_^2^	8.973
PB(*α*) – PB(*α*+1)	100 *k*_B_ *T*/*r*_0_^2^	3.38
PBP – DBP	1	41.59
DBP – DBP	1	33.8

The bond angle potential for the CG-DNA molecules is given by
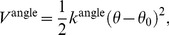
(5)where *k* ^angle^ is the force constant, *θ* is the bond angle, and *θ*_0_ is the equilibrium bond angle. The stiffness of bond angles formed by adjacent three PB beads in the backbone is directly related to the persistence length of DNA [28]. All bond angles and force constants are listed in Table 3. The listed parameters for PB(*α*) – PB(*α*+1) – PB(*α*+2) correspond to the typical persistence length of DNA, 50 nm [28].

**Table 3:** Parameters used in the bond angle energy of the CG model.

Angle type	*k* ^angle^ (kcal/mol)	*θ*_0_ (degree)
PP(*α*) – PB(*α*) – PB(*α*+1)	100	90
PB(*α*) – PB(*α*+1) – PP(*α*+1)	100	90
PB(*α*−1) – PB(*α*) – PB(*α*+1)	87.7	32.7

Torsion angles defined by PP(*α*) – PB(*α*) – PB(*α*+1) – PP(*α*+1) are restrained by
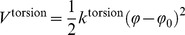
(6)where *k* ^torsion^ is the force constant, *φ* is the torsion angle, and *φ*_0_ is the equilibrium torsion angle. All torsion angles and force constants are listed in Table 4.

**Table 4:** Parameters used in the torsional angle energy of the CG model.

Torsion type	*k* ^torsion^ (kcal/mol)	*φ*_0_ (degree)
PP(*α*) – PB(*α*) – PB(*α*+1) – PP(*α*+1)	131.6	36

Excluded volume effects are described by a half-harmonic potential,
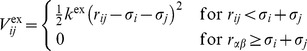
(7)where *k* ^ex^ is the force constant. For electrostatic interactions between beads, the effective Yukawa pair-potential of DLVO (Derjaguin, Landau, Verwey, and Overbeek) theory was used and is given by [29]

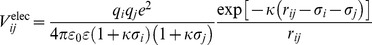
(8)where *e* is the elementary charge (4.803×10^−10^ esu), *ε*_0_ is the permittivity of the vacuum, *ε* is the dielectric constant of the medium, and *κ* is the inverse of the Debye screening length. It is worth to noting that *q* in Eq. (8) is the effective charge, which is parameterized to change binding affinities between the modeled CG-protein and CG-DNA based on an umbrella sampling technique as described in the Umbrella sampling section of METHODS. Bead pairs that satisfy |*i*−*j*|≤4 are excluded in calculating the non-bonded interactions *V* ^ex^ and *V* ^elec^.

### Simulation conditions

BD simulations were performed under non-periodic boundary conditions. The simulation temperature was set to 298 K, and the time step was set to 0.25 ps. The diffusion tensor estimated by the RPY tensor and its Cholesky decomposition for computing Brownian displacement satisfying the fluctuation-dissipation theorem were updated every 200 steps. BD simulations were performed for 25 µs. Coordinates and energies were sampled every 10,000 steps (2.5 ns). The dielectric constant of the medium *ε* was set to 78.5. The Debye length 1/*κ* was set to 7.8 Å, which corresponds to a NaCl concentration of 0.15 M at 298 K. *k* ^ex^ was set to 1 kcal/mol/Å^2^. A cutoff distance of 40 Å was used for the non-bonded interactions *V* ^ex^ and *V* ^elec^. The DNA length was 200 bp, in which our CG model has 200 pseudo residues consisting of 200 PP and 200 PB beads.

The geometrical center of the CG-DNA model was placed at the origin of the Cartesian coordinates, and the longest axis of the DNA was placed along Z-axis. The CG-protein was placed just above the DNA molecule. To study the effects of HI on the 1D sliding of the CG-protein model, we performed two different types of BD simulations: one with full HI; that is, HI within each CG-protein and DNA molecules as well as between CG molecules are considered. The other is a simulation with only intramolecular HI, where intermolecular HI are neglected. Hereafter, we call the former “with inter-HI” and the latter “without inter-HI”. We also considered two different treatments for CG-DNA for analyzing effects of DNA flexibility on 1D sliding of the CG-protein: one is “restrained CG-DNA”, where all beads of the CG-DNA molecule are restrained at their initial positions by a harmonic potential with a force constant of 1 kcal/mol/Å^2^. The other is “flexible CG-DNA”, where two PP and PB beads at both termini are restrained at their initial positions by a harmonic potential with a force constant of 0.01 kcal/mol/Å^2^. For each condition, ten independent BD simulations were performed with different random seeds.

### Umbrella sampling

An umbrella sampling method was employed for estimating the binding free energy of the CG-DNA binding protein, with various charges on the DBP beads, to DNA [30]. The geometrical center of the CG-DNA model was placed at the origin of the Cartesian coordinates, and the longest axis of the DNA was placed along Z-axis. All beads of the DNA were restrained in Cartesian space by a harmonic potential throughout the umbrella sampling simulations. The PBP bead of the CG-protein was placed on the X-axis at a distance of 90 Å as its initial position. PBP beads are allowed to move only in the X-Y plane by applying a harmonic potential along the Z-axis with a force constant of 1 kcal/mol/Å^2^. An umbrella potential *V* = 1/2*k* ^umb^(*r*−*r*_0_)^2^ was applied between the PBP and a PB bead nearest the origin with *r*_0_ = 90, 89, ···, 31 Å (total 60 windows) and *k* ^umb^ = 5 kcal/mol/Å^2^. For each *r*_0_, a 25 ns BD simulation was performed, where the first 5 ns are for equilibration and the rest for sampling. The potential mean force was constructed by WHAM [31]. The umbrella sampling BD simulations and their analysis were performed 5 times with different random seeds.

### Estimation of 3D diffusion coefficients and Stokes radii of the model proteins by rigid-particle theory

Rigid-particle theory is a well-known method to compute diffusion properties, such as the translational and rotational diffusion coefficients at infinite dilution, *D*_3D_^T^ and *D*_3D_^R^, respectively, of rigid objects constructed from many particles [32]–[34]. The method gives diffusion coefficients of the object very close to the values estimated from BD simulations [35], [36]. In this study, the Stokes radii of the model CG-protein *a*_pro_ were estimated via the Stokes-Einstein equation connecting the Stokes radius and translational and rotational diffusion coefficients, given by
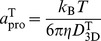
(9)and
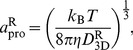
(10)respectively. Thus, two estimates of the Stokes radii are provided.

## Results/Discussion

### Binding affinity estimated by umbrella sampling

**Figure 2:**
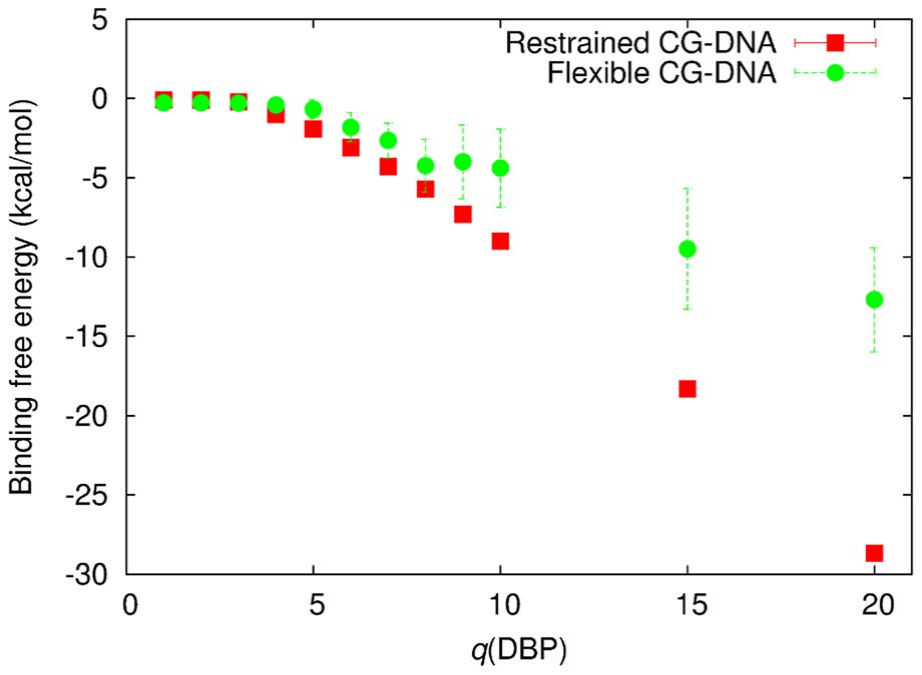
Binding free energy estimated by the umbrella sampling method for various charge values of DBP beads, *q*(DBP), in the CG-protein model with the restrained and flexible CG-DNA models. Stokes radius of the PBP bead, *a*(PBP), of 40 Å was used for this estimation.

Binding affinities for non-specific DNA binding for several proteins have been estimated to be in the range of 10 *k*_B_ *T* (5.9 kcal/mol) to 15 *k*_B_ *T* (8.9 kcal/mol) at physiological salt concentrations [37]. We employed the umbrella sampling technique to estimate the binding affinities of the CG model with various charges of the DBP beads, *q*(DBP). In Fig. 2, binding free energies as a function of *q*(DBP) are shown. CG-proteins with *q*(DBP) = 8–10 with restrained CG-DNA have a binding free energy of 5.71±0.02 to 9.00±0.02 kcal/mol, which is close to the experimental estimate. For the flexible CG-DNA model, fluctuation of the CG-DNA reduces binding affinity; here CG-proteins with *q*(DBP) = 8 to 15 have binding free energies within the range of experimental estimates.

### Comparison with theory

In order to compare our simulation results to the theory, we first performed BD simulations using *q*(DBP) = 20 with the restrained CG-DNA model in the absence of inter-HI. With this *q*(DBP), the CG-protein virtually never detaches from DNA during the simulation length due to the protein's very high binding affinity of −30 kcal/mol. In our model, all PP beads have the same charge, and the DNA sequence effect is not considered. Therefore, it is possible that the energy landscape along DNA might be very smooth. These conditions make the simulation closely correspond to the model in the BBX theory. The binding affinity −30 kcal/mol is not biologically relevant. However, our purpose in this section is to compare BD results with the BBX theory. In subsequent analysis, *R*oc is defined as the distance between the DNA axis and the center of diffusion of the CG-protein calculated by the rigid-particle theory.

**Figure 3:**
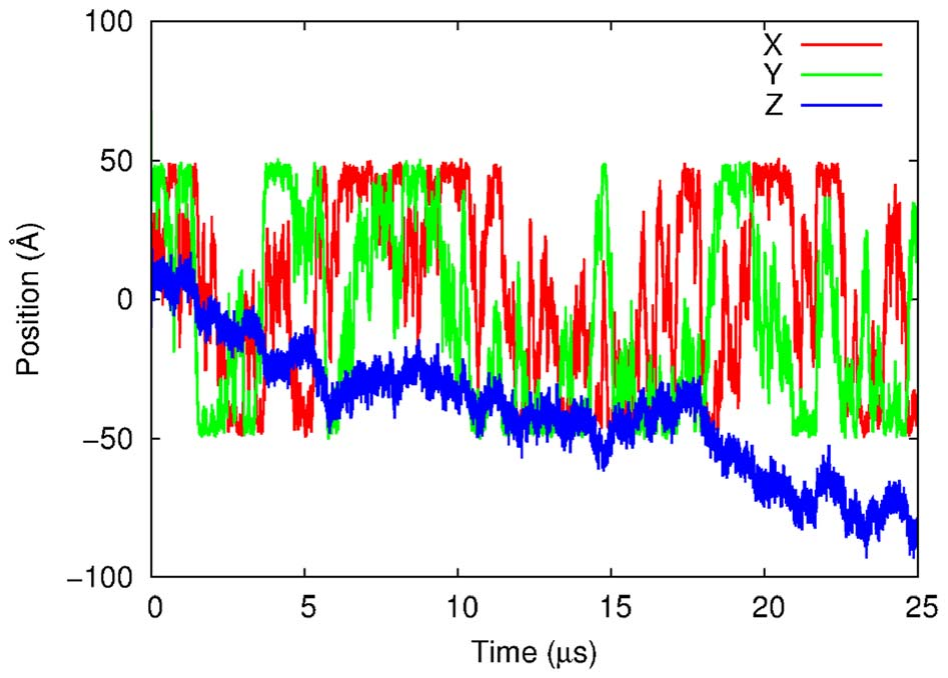
Representative trajectories of X, Y, and Z positions of the PBP bead in the CG-protein molecules with *a*(PBP) of 40 Å and *q*(DBP) of 20. The BD simulation was done in the absence of intermolecular HI and all beads of the CG-DNA molecule were restrained by a harmonic potential.

In Fig. 3, a representative trajectory of the model CG-protein with *q*(DBP) = 20 and *a*(PBP) = 40 Å is shown (see also Movie S1). For this condition, the CG-protein showed rotation-coupled sliding along Z-axis as expected without detaching from the model CG-DNA for all *a*(PBP) ( = 30, 40 and 50 Å) values examined. The diffusion coefficients and related properties or proteins of various sizes estimated from the BD simulations in the absence of inter-HI and the rigid particle theory are listed in Table 5. In this table, the protein's Stokes radii were estimated by Eq. (9), and used for estimating *D*_1D_^theory^ via Eq. (2). *D*_1D_ are much smaller than *D*_3D_ by a factor of ∼100. This large reduction in diffusion rate is qualitatively consistent with the theoretical expectation from the BBX model. However, *D*_1D_^theory^ values estimated with *a*_pro_^T^ are smaller than *D*_1D_ calculated by the BD simulations, which is especially evident for larger *a*(PBP).

**Table 5:** Diffusive properties of the CG-DNA binding protein obtained from BD simulations in the absence of intermolecular HI with the fixed CG-DNA model and given by the BBX theory.^a^

*a*(PBP)	*a*_pro_^T^	*D*_3D_	*D*_1D_	*R*_OC_	*D*_1D_^theory^	*D*_1D_/*D*_1D_^theory^
30	31.5	7.79	0.086±0.036	40.4±1.1	0.074	1.16
40	40.5	6.06	0.063±0.021	44.0±1.1	0.041	1.54
50	50.1	4.89	0.057±0.020	45.8±1.1	0.025	2.28

a Units in Å for radii and *R*oc, and Å^2^ ns^−1^ for diffusion coefficients, respectively, are used.

To investigate the origin of this deviation, Stokes radii of proteins were also estimated from rotational diffusion coefficients computed by the rigid-particle theory using Eq. (10), which are listed in Table 6. Although *a*_pro_^T^ values are close to *a*(PBP), *a*_pro_^R^ are almost a constant 20 Å for all protein sizes, which is much smaller than *a*(PBP) as well as *a*_pro_^T^ values. If a given object is completely spherical, like the protein model in BBX theory, *a*_pro_^R^ should be equal to *a*_pro_^T^. This discrepancy of *a*_pro_^R^ in between our model and the BBX theory may give rise to the deviation in 1D diffusion coefficient. To check this possibility, we check if *D*_1D_^theory^ gives a value close to *D*_1D_ when both *a*_pro_^T^ and *a*_pro_^R^ values are used in estimating *D*_1D_^theory^. As explained in Introduction, the denominator in Eq. (2) is the sum of translational and rotational friction contributions. So, we re-estimate the 1D diffusion coefficients using *a*_pro_^T^ and *a*_pro_^R^ values as follows:
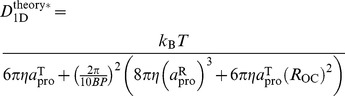
(11)The difference of Eq. (11) from Eq. (2) used for calculating *D*_1D_^theory^ listed in Table 5 is the use of *a*_pro_^R^ value in the second term in the denominator. The corrected 1D diffusion coefficients, *D*_1D_^theory*^ listed in Table 6, for *a*(PBP) = 40 and 50 Å are much closer to the *D*_1D_ values directly computed from the BD simulations. This result indicates that the small *a*_pro_^R^ values compared to *a*_pro_^T^ in our model result in larger *D*_1D_ than the theoretical estimates from the original *D*_1D_^theory^ of Eq. (2).

**Table 6:** Diffusive properties using corrected values.^a^

*a*(PBP)	*a*_pro_^T^	*a*_pro_^R^	*D*_1D_^theory*^	*D*_1D_/*D*_1D_^theory*^
30	31.5	20.4	0.109	0.82
40	40.5	20.6	0.076	0.91
50	50.1	20.6	0.056	0.96

a Units in Å for radii and *R*oc, and Å^2^ ns^−1^ for diffusion coefficients, respectively, are used.

*D*_1D_/*D*_1D_^theory^ and *D*_1D_/*D*_1D_^theory*^ increase with *a*(PBP). For *a*(PBP) = 30 Å, the correction does not improve the result and even makes it slightly worse. The distance between PBP and DBP beads is 41.6 Å (Table 2) and *a*(DBP) = 8 Å (Table 1). Therefore, the Stokes radii of PBP and DBP overlap if *a*(PBP) is larger than 33.6 Å. The RPY tensor described in Eq. (3) is defined for overlapping particles, which was derived to remain the tensor positive definite. However, the physical meaning of this form is problematic [38]. The increase on *D*_1D_/*D*_1D_^theory^ and *D*_1D_/*D*_1D_^theory*^ values with Stokes radius may be due to particle overlap and use of the RPY tensor for overlapping particles. For *a*(PBP) = 30 Å, PBP and DBP do not overlap. For *a*(PBP) = 30 Å, although PBP and DBP do not overlap, *D*_1D_^theory^ and *D*_1D_^theory*^ values slightly deviate from *D*_1D_ obtained from BD simulations. The deviation may be rooted in the shape difference of modeled proteins; BBX theory assumes proteins are completely spherical objects, but our CG-protein models are not completely spherical.

### Effects of HI between protein and DNA, and DNA flexibility on 1D sliding speed

In this section, we try to evaluate the effects of HI between the CG-protein and CG-DNA, and DNA flexibility on 1D diffusion. In Table 7, the apparent 1D diffusion coefficients of proteins with a *q*(DBP) of 20 estimated by the BD simulations in the presence and absence of inter-HI, and with the restrained and flexible CG-DNA are listed. A representative trajectory of CG-protein sliding dynamics on flexible DNA in the absence of inter-HI is shown in Movie S2. Inter-HI reduce the 1D diffusivity by 30% and 40% on average over three different CG-protein sizes for both the restrained and flexible CG-DNA, respectively. Thus, the implication is that inter-HI effects are quite robust and insensitive to details. This reduction could be explained by correlated motions between the CG-protein and DNA models caused by inter-HI. The flexibility of CG-DNA increases 1D diffusivity by factors of 2.6 and 2.1 on average over three different CG-protein sizes in the absence and presence of inter-HI, respectively. This mechanism will be discussed below.

**Table 7:** Apparent 1D diffusion coefficients (Å^2^ ns^−1^) of proteins with *q*(DBP) of 20 estimated by BD simulations with the restrained and the flexible CG-DNA model in the presence and absence of intermolecular HI.

	Restrained CG-DNA		Flexible CG-DNA	
*a*(PBP) (Å)	W/O intermolecular HI	W/intermolecular HI	W/O intermolecular HI	W/intermolecular HI
30	0.086±0.036	0.068±0.028	0.203±0.060	0.139±0.045
40	0.063±0.021	0.047±0.009	0.180±0.075	0.096±0.038
50	0.057±0.020	0.038±0.009	0.146±0.042	0.083±0.032

### Effect of binding affinity of the protein on its 1D sliding

**Figure 4:**
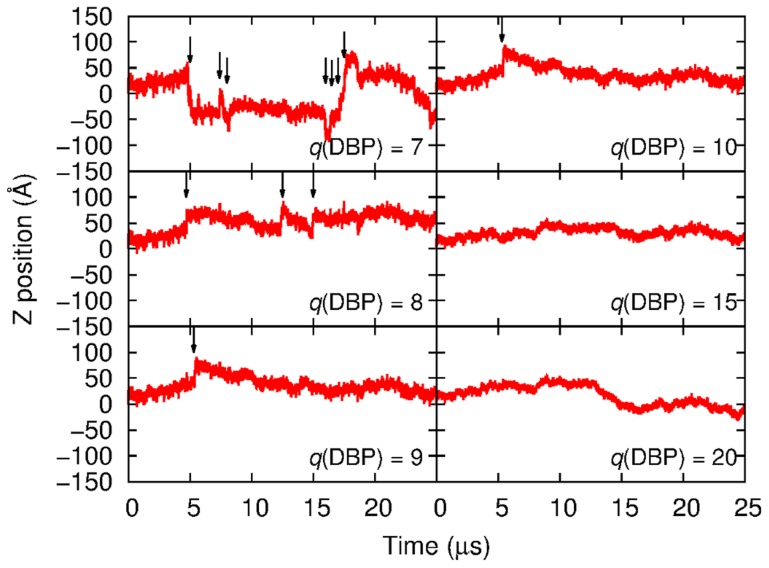
Representative trajectories of z position of the PBP bead in the CG-protein molecules with *a*(PBP) of 40 Å and *q*(DBP) of 7, 8, 9, 10, 15, and 20. BD simulations shown in this figure were done in the absence of intermolecular HI using the same random seed. Arrows indicate times that hopping was observed.

**Figure 5:**
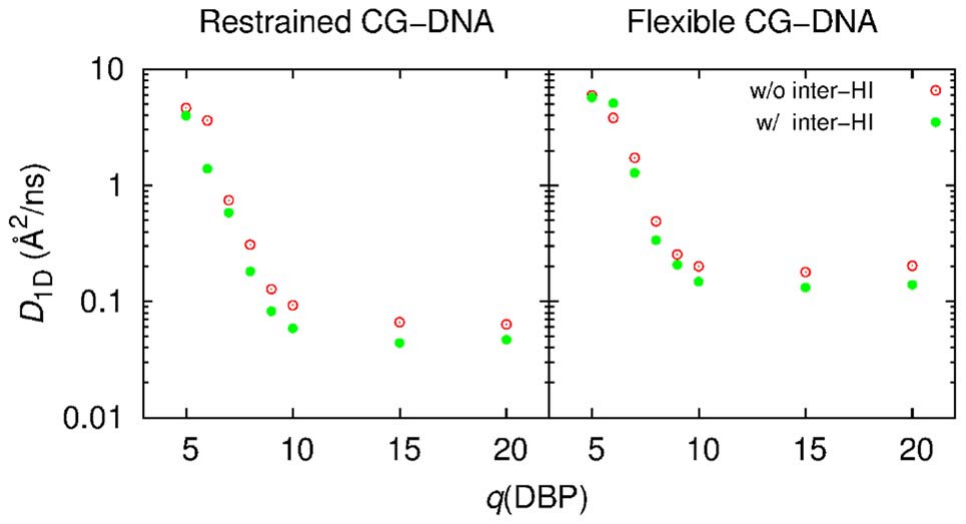
Apparent 1D diffusion coefficients of the CG-protein molecules with *a*(DBP) = 40 and *q*(DBP) = 5, 6, 7, 8, 9, 10, 15, and 20 obtained from the BD simulations (left) with the restrained CG-DNA and (right) with the flexible CG-DNA models in the presence and absence of intermolecular HI. These values are average over ten BD simulations.

Finally, the effects of charge *q*(DBP) on 1D sliding rate are examined. In Fig. 4, representative trajectories in Z position of the model CG-proteins with various *q*(DBP) and *a*(PBP) = 40 Å are shown, where the restrained CG-DNA molecules were used. During 25 µs BD simulations, the CG-protein with *q*(DBP) of 7 to 10 hopped along the CG-DNA molecule (see Movie S3 that shows hopping of the CG-protein). This hopping is prominent for smaller *q*(DBP) values. This trend was not changed for the simulations with the flexible CG-DNA model. Apparent 1D diffusion coefficients of the CG-protein with *a*(PBP) = 40 Å and various *q*(DBP) with the restrained and flexible CG-DNA in the presence and absence of inter-HI are shown in Fig. 5. The CG-proteins with smaller *q*(DBP) values (<10) tend to slide quickly due to hopping for both CG-DNA models. For *q*(DBP)>10, *D*_1D_ reached the same lower bound for both CG-DNA models. CG-proteins with *q*(DBP) = 8 to 10 have binding affinities within experimental estimates of non-specific DNA binding. Those proteins in our model showed hopping along DNA. Direct observations of the hopping by single molecule experiments are currently very difficult due to the limited experimental resolution. Our simulation supports the possibility of hopping for non-specifically DNA bounded proteins initially envisioned in the theory.

Reduction due to inter-HI was observed for the whole range of *q*(DBP), which was evident for the larger *q*(DBP). Acceleration by DNA flexibility was also seen for whole range of *q*(DBP) values, by about a factor of 2. The CG-proteins with *a*(PBP) = 30 Å and 50 Å show almost similar results for the reduction by inter-HI and acceleration by fluctuations. Regardless of inter-HI, 1D diffusion for the CG-protein with *q*(DBP) = 15 and 20 in the system with flexible CG-DNA are 2 to 3 times faster than those with same charge values as well as for *q*(DBP) = 10 in the restrained CG-DNA model. However, CG-protein binding affinity with *q*(DBP) = 10 in the restrained CG-DNA is lower than that with *q*(DBP) = 15 and 20 in the flexible CG-DNA system as shown in Fig. 2. Local and temporary steric repulsion between the CG-protein and CG-DNA model caused by fluctuations of the DNA molecule may enhance the rate of 1D sliding.

### Implications of simulation results to experiments and limitations of the CG-model

Free energy roughness along the sliding path caused by the sequence-dependent atomistic interactions, e.g. hydrogen bonding, is also considered to be an important factor for reducing 1D diffusivity of the DNA bound protein. This effect is often evaluated by the Zwanzig formula for diffusion in a rough potential in 1D space, in which the reduction factor is written as exp[(−*ε*/*k*_B_ *T*)^2^], with energy roughness *ε* [39]. This reduction is very sensitive to *ε*. From the Zwanzig formula and experimental results on apparent diffusion coefficients of several DNA binding proteins, *ε* was estimated 1.1±0.2 *k*_B_ *T* [7], which means a reduction factor of 0.3. In this analysis, all sources of reduction except for helical diffusion are assumed to be due to energy roughness. However, as seen in this work, inter-HI decrease and DNA fluctuation increase the diffusivity of proteins along DNA

Here, the RPY tensor was employed to represent HI between the CG-protein and CG-DNA models. However, the RPY tensor only includes the far-field part of hydrodynamic effects [23], [24]. A recent simulation study of the association of two non-polar model objects clearly showed that at short distances (<1–2 nm) molecular scale effects dominate, giving rise to deviations from continuum hydrodynamic theory [40]–[42]. Even though a more sophisticated hydrodynamic model was used, the deviation from the atomistic simulation results was not eliminated. Therefore, we may need to find a better description of HI at short distances. However, we believe that our simulation results using the RPY tensor still provide the important qualitative features of HI. Finally, our BD simulations show the possibility that DNA structural fluctuations enhance 1D diffusion of DNA binding proteins by steric collisions between the protein and DNA. However, since proteins are also flexible, this effect may be damped.

### Conclusions

In this work, we have developed CG models of DNA binding protein and DNA for dissecting the sliding mechanisms of a protein along the DNA. By considering HI we could compare our simulation results with the theoretical model of rotation coupled sliding along helical path of DNA proposed by Bagchi, Blainey, and Xie [17]. This makes it possible to elucidate the relative importance of hydrodynamic forces between the protein and DNA, DNA flexibility, and binding affinity of the protein to the DNA on 1D diffusivity of the protein along the DNA. Our simulations under conditions similar to the BBX model showed that 1D diffusivity obtained from the BD simulations using our model are 1.2–2.2 times faster than theoretical estimates. This discrepancy is mainly due to a low rotational friction of the CG-protein in our model compared to the theoretical treatment in BBX model. Second, BD simulations with intermolecular HI represented by the RPY tensor show that HI reduce 1D diffusivity by 30%. Third, a CG-protein whose binding affinity to CG-DNA is in the range of experiment, −5 to −10 kcal/mol, shows hopping along DNA. This results in an increase of its apparent 1D diffusion coefficients. Direct observations of the hopping by single molecule experiments are currently very difficult due to the limited time and space resolutions in experiments.

The model developed in this work is quite simple, but still we can do “experiments” to elucidate the sliding mechanisms of DNA binding proteins by changing several parameters. Early BD simulation work on protein diffusion in concentrated DNA solutions using a CG model reasonably well reproduce experimental results [43], where the protein and DNA are represented one sphere and strings of beads, respectively, and electrostatic interactions are calculated from a Debye-Hückel potential. This work also shows usefulness of a very simple CG model to analyze the dynamics of macromolecules. In this work, we have only changed the effective charge parameter of the CG-DNA binding protein to reproduce the biologically relevant binding affinity. However, since HI are long-range effects and the dominant effect is insensitive to shape of the molecule, we believe that effects of HI on protein sliding observed in our BD simulation would be seen in the simulations with different particle radii and higher-resolution models.

Inclusion of HI in BD simulations is often a computational bottleneck to large-scale simulations. To overcome this difficulty, we developed a Krylov subspace method for computing correlated Brownian noise vectors which scales as *O*(*N* ^2^) with an *N* particle system, whereas an *O*(*N* ^3^) computation is required in a conventional BD algorithm [44]. The Krylov method with a particle-mesh Ewald method, which is based on fast-Fourier transform for computing hydrodynamic effects, enables BD simulations with *O*(*N*log*N*) scaling in computation and *O*(*N*) memory storage [45]. Combining these advance BD algorithms and the CG model developed here would give us a possibility to perform large-scale BD simulations of DNA binding proteins in a crowded intracellular environment, which should enable a deeper understanding of the experimental results. These simulations are currently underway.

## Supporting Information

Video S1 **BD simulation of sliding of the CG-protein along the restrained CG-DNA in the absence of intermolecular hydrodynamic interactions.** This trajectory corresponds to Fig. 3 in the main text. The Stokes radius of the PBP bead of the CG-protein, *a*(PBP), is 40 Å and the charge at *q*(DBP) of the CG-protein is 20. In this movie, the beads' radii correspond to their excluded volume radii.
(MP4)

Video S2 **BD simulation of the sliding of the CG-protein along flexible CG-DNA in the absence of intermolecular hydrodynamic interactions.** The Stokes radius of the PBP bead of the CG-protein, *a*(PBP), is 40 Å and the charge at *q*(DBP) of the CG-protein is 20. In this movie, the beads' radii correspond to their excluded volume radii.
(MP4)

Video S3 **BD simulation of the sliding of the CG-protein along the restrained CG-DNA in the absence of intermolecular hydrodynamic interactions.** This movie corresponds to the trajectory shown at top left panel in Fig. 4 of the main text. The CG-protein, which has *a*(PBP) of 40 Å and *q*(DBP) of 7, shows hopping along the CG-DNA. In this movie, beads' radii correspond to their exclude volume radii.
(MP4)
